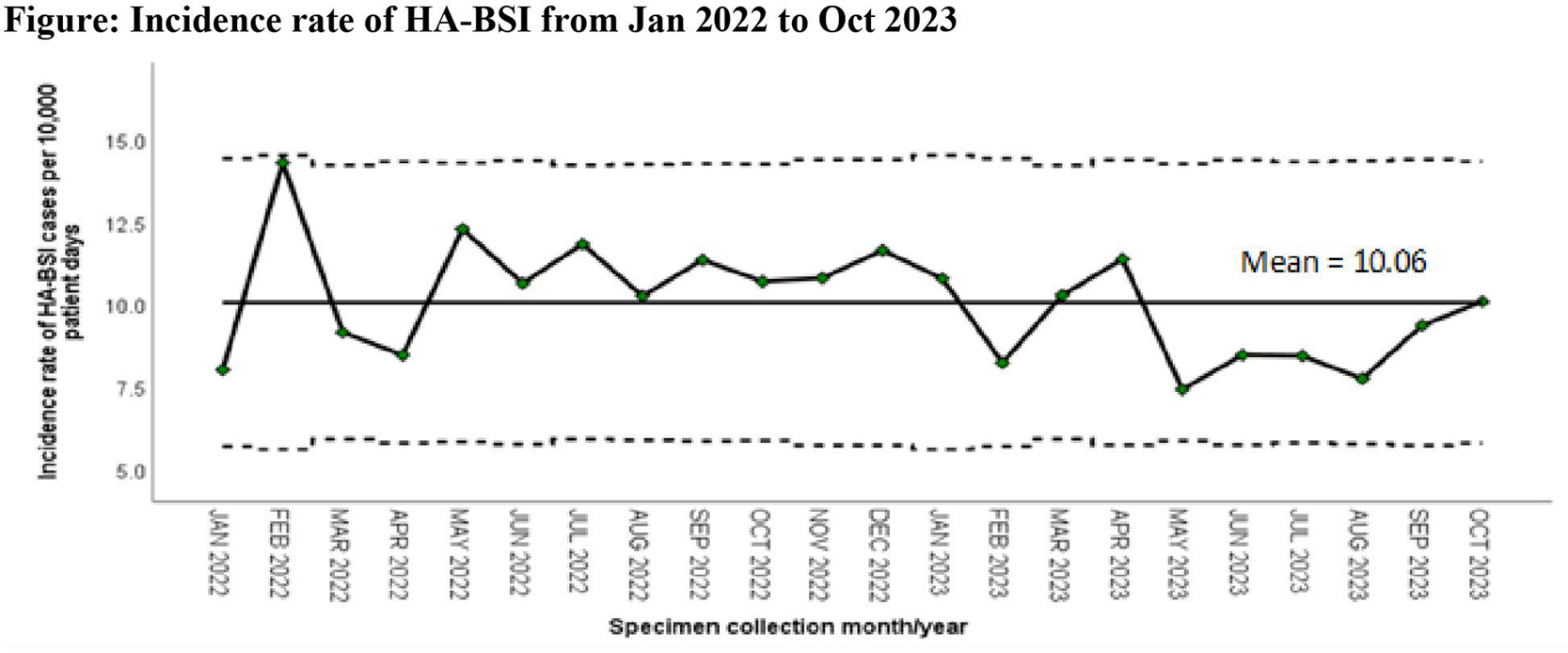# Comparative Analysis of Healthcare-associated Bloodstream Infections & CLABSI Surveillance in A Singaporean tertiary Hospital

**DOI:** 10.1017/ash.2024.334

**Published:** 2024-09-16

**Authors:** Shalvi Arora, Sheena Jin Min Ong, Pinhong Jin, Aung Myat Oo, May Kyawt Aung, Yak Weng Darius Chan, Yang Yong, Ian Wee Liang En, Xiang Ying Jean Sim, Lai Chee Lee, Moi Lin Ling, Indumathi Venkatachalam

**Affiliations:** Singapore General Hospital; Singhealth

## Abstract

**Background:** Healthcare-associated central line associated bloodstream infection (HA-CLABSI) surveillance is important for monitoring healthcare-associated infections (HAIs) and evaluating effectiveness of infection prevention (IP) measures. However, implementing it is a laborious and time-consuming approach. Exclusive focus on central lines neglects HAI risk due to peripheral vascular catheters. This study aimed to assess whether HA-CLABSI incidence could be inferred from HA-bloodstream infection (BSI) trends and explore shift to HA-BSI surveillance. **Methods:** The study was performed in a Singaporean tertiary care hospital. Electronic medical records review was performed to determine whether positive blood cultures met Centers for Disease Control/National Health Safety Network (CDC/NHSN) definitions for HA-CLABSI and HA-BSI. Incident episodes of HA-BSI were included (excluding positive cultures repeated within 14 days). Incident organisms were explored to identify common causative pathogens (excluding same organisms isolated from cultures repeated within 14 days). CLABSI and BSI occurring ≥72hrs after admission were considered healthcare-associated. Patients under oncology or hematology service were considered immunocompromised. Incidence rates (IR) per 10,000 patient-days, patient characteristics and causative pathogens were compared between both indicators. **Results:** From January 2022 to October 2023, mean IR for HA-CLABSI was 0.63 (n=68) and for HA-BSI was 10.06 (n=1094). Median age of patients with HA-CLABSI was 66 years and HA-BSI was 68 years. HA-CLABSI and HA-BSI were more common in males (60.86% & 58.68%). Median duration between admission to HA-CLABSI was 20 days and to HA-BSI was 12 days. Median duration between central line insertion to HA-CLABSI was 16 days. Of 1094, 631 (57.7%) patients had vascular catheter(s) (i.e., IV cannula, port-a-cath, peripherally-inserted central catheter or central line) inserted at time of HA-BSI diagnosis, of whom 46 (7.3%) patients had CLABSI ±2days from positive blood culture. There was no significant correlation between monthly aggregate data from these indicators (Spearman’s correlation coefficient= 0.36, p-value=0.1). Predominant organisms causing HA-CLABSI and HA-BSI were gram negative bacteria (GNB, 40% & 57.21%), gram positive bacteria (24.71% & 22.23%), and fungi. Common GNB in CLABSI patients were Pseudomonas spp. and Stenotrophomonas maltophilia (8.24%), followed by Serratia marcescens and Klebsiella pneumoniae (5.88%). The frequent GNB in HA-BSI patients were Escherichia coli (15.4%), Klebsiella pneumonia (12.68%), and Pseudomonas spp. (6.69%). Common multi-drug resistant organisms were vancomycin-resistant Enterococcus faecium (10.59% & 3.69%) and methicillin-resistant Staphylococcus aureus (10.59% & 3.07%). **Conclusion:** HA-BSI did not correlate with HA-CLABSI. HA-BSI reflects heterogenous population outcomes. For utilization as surveillance indicator, further assessment on exclusion criteria is required to improve specificity.

**Figure:**
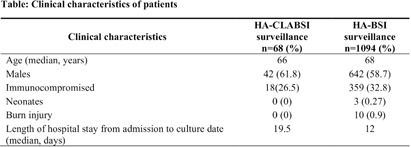

**Figure:**
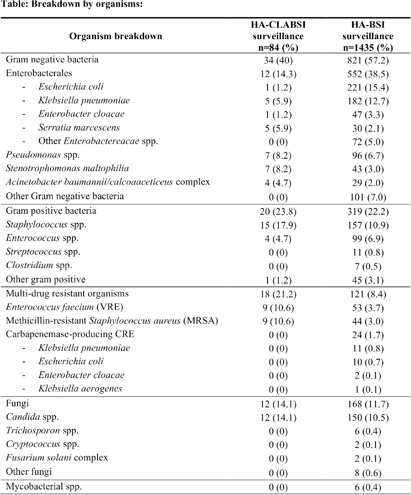

**Figure:**
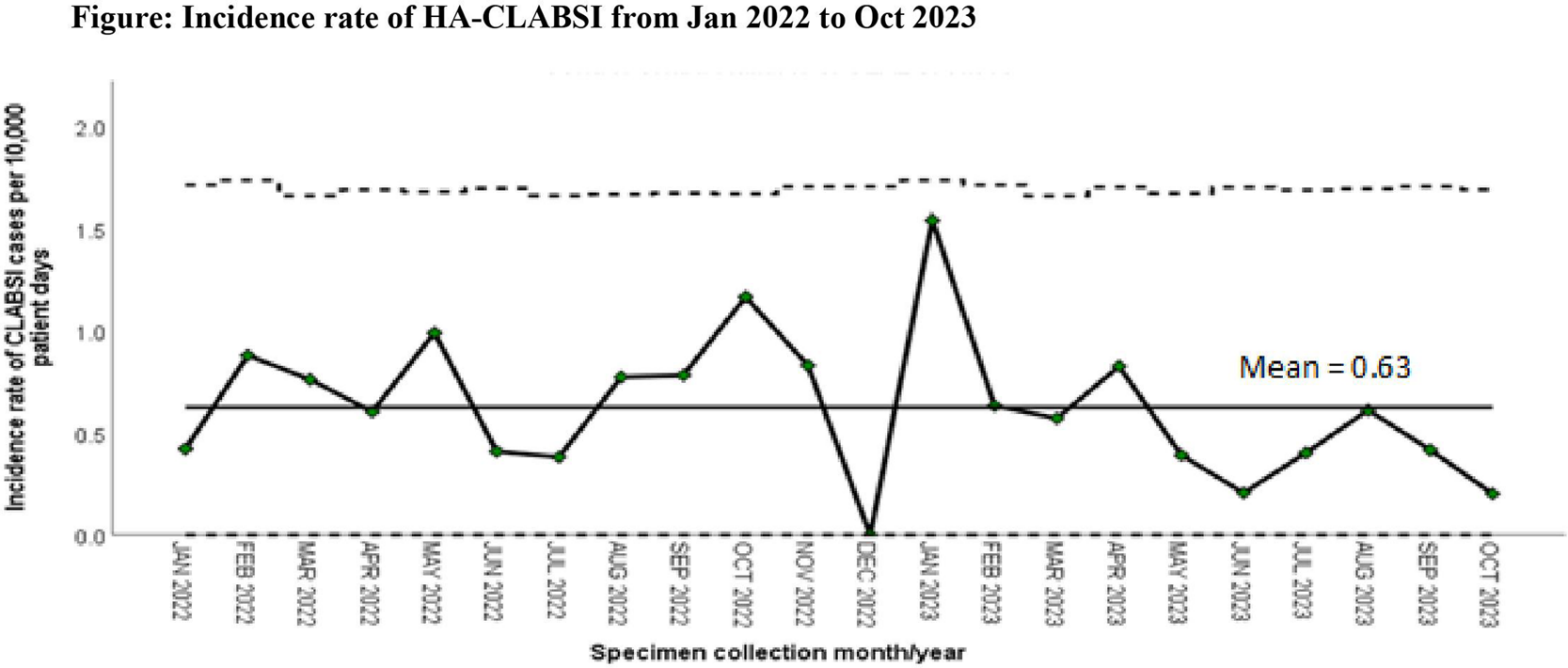

**Figure:**